# Artificial Humic Acid Mediated Carbon–Iron Coupling to Promote Carbon Sequestration

**DOI:** 10.34133/research.0308

**Published:** 2024-02-19

**Authors:** Yibo Lan, Shuang Gai, Kui Cheng, Zhuqing Liu, Markus Antonietti, Fan Yang

**Affiliations:** ^1^ School of Water Conservancy and Civil Engineering, Northeast Agricultural University, Harbin 150030, China.; ^2^ International Cooperation Joint Laboratory of Health in Cold Region Black Soil Habitat of the Ministry of Education, Harbin 150030, China.; ^3^ College of Engineering, Northeast Agricultural University, Harbin 150030, China.; ^4^ Department of Colloid Chemistry, Max Planck Institute of Colloids and Interfaces, 14476 Potsdam, Germany.

## Abstract

Fe (hydr)oxides have a substantial impact on the structure and stability of soil organic carbon (SOC) pools and also drive organic carbon turnover processes via reduction–oxidation reactions. Currently, many studies have paid much attention to organic matter–Fe mineral–microbial interactions on SOC turnover, while there is few research on how exogenous carbon addition abiotically regulates the intrinsic mechanisms of Fe-mediated organic carbon conversion. The study investigated the coupling process of artificial humic acid (A-HA) and Fe(hydr)oxide, the mechanism of inner-sphere ligands, and the capacity for carbon sequestration using transmission electron microscopy, thermogravimetric, x-ray photoelectron spectroscopy, and wet-chemical disposal. Furthermore, spherical aberration-corrected scanning transmission electron microscopy–electron energy loss spectroscopy and Mössbauer spectra have been carried out to demonstrate the spatial heterogeneity of A-HA/Fe (hydr)oxides and reveal the relationship between the increase in Fe-phase crystallinity and redox sensitivity and the accumulation of organic carbon. Additionally, the dynamics of soil structures on a microscale, distribution of carbon–iron microdomains, and the cementing-gluing effect can be observed in the constructing nonliving anthropogenic soils, confirming that the formation of stable aggregates is an effective approach to achieving organic carbon indirect protection. We propose that exogenous organic carbon inputs, specifically A-HA, could exert a substantial but hitherto unexplored effect on the geochemistry of iron–carbon turnover and sequestration in anoxic water/solid soils and sediments.

## Introduction

Soils are the most extensive carbon reservoir in terrestrial ecosystems, with worldwide estimates varying from 2,344 PgC to almost 3,000 PgC [1]. Iron (Fe) has a critical role in “capturing” and “forming rust sinks” among other factors that contribute to organic carbon (OC) sequestration. Besides, Fe (hydr)oxides adsorb OM on its surface and/or act as catalysts for organic reactions, which in turn changes the surface properties and crystal phases [2,3]. In particular, Fe(III) (hydro)oxides undergo reductive dissolution in anoxic environments, releasing Fe(II), which may remain in solution and precipitate as secondary Fe(II) or mixed-valent Fe minerals, or be reoxidized by biotic or abiotic processes to form mixed-valent Fe(II/III) or Fe(III) minerals [3,4]. The fact that the protection provided by Fe (hydr)oxides favors the persistence of iron–carbon complexes in soils and sediments for hundreds or even thousands of years has been well documented [3,5,6]. However, the general assumption that Fe (hydr)oxides provide a protective function for organic matter (OM) is simplistic, and investigations into the spatial and functional complexity of Fe (hydr)oxides–OM interactions are still in limbo.

In soils and sediments, Fe (hydr)oxides–OM associations play a key role in the structuring and compartmentalizing of the abiotic/biotic reaction space into microsites. In addition to this, Fe (hydr)oxides and OC can act as either the core of a building unit, a cementing agent, or a gluing agent to form soil aggregates [7]. This means that surface properties, such as electrical charge, allow OM and Fe (hydr)oxides to adhere together to form microstructured clay- and silt-sized composite particles. These composite particles then act as composite building units, including so-called “organo-mineral associations”, nanoscale organo-mineral composites, as part of “hypothetical building units" or “mineral-organic associations” [7]. According to studies, the OC-Fe component in soil aggregates constitutes 30% to 50% of the total OC, with exogenous inputs capable of pushing the OC-Fe proportion beyond 80% [8]. Siebers et al. [9]showed that lacking OM reduced microaggregate stability and resulted in releasing removable colloids. Hernandez-Soriano et al. [10] demonstrated the importance of organo-mineral interactions in influencing the stability of total C, especially for aliphatic C, supporting the hypothesis that microaggregates are formed through interactions via organo-mineral. Nevertheless, the majority of previous studies focused on the adsorption and transport of organic matter accompanied with Fe(III) (hydro)oxides. The iron cycling in stable OC storage and the regulation of OC by iron mineral redox have yet to be comprehensively assessed, particularly regarding the positive contribution of biomass-based exogenous carbon (EOC) input from biomass on iron–carbon biogeochemical cycling.

The incorporation of EOC into soil to form stable soil organic carbon (SOC) is thereby an essential nature-inspired solution for mitigating carbon loss [11]. Various strategies have been proposed to stabilize carbon in the soil, including straw return, conservation tillage (reduced or no tillage) and rock residue. According to a 3-decade study, increasing crop residue/straw return practices have contributed markedly to carbon sequestration in Chinese farmland. However, straw biomass is not a stable soil carbon sequestrant [12]. Artificial humic acid (A-HA), an emerging EOC derived from biological by-products or waste, has a carbon-negative property [13,14]. Our previous research has identified A-HA as a facilitator for the establishment of interactive microbial systems along mineral particle interfaces and plays an important role in maintaining soil ecosystem services [15,16]. A-HS has been proved to change soil structure, support water and mineral binding, avoid fertilizer mineralization, and furthermore support plant growth [14–17]. We emphasized the role of A-HA as a soil microbial carbon pump based on results that A-HA markedly promotes the growth of *Rubbrivivax gelatinosus*, which has multiple roles in carbon sequestration, and modulates the microbial community structure, resulting in soil carbon sequestration and reduction of OC loss [17,18].

Given that the aforementioned “rusty carbon sinks” in A-HA-rich soil systems focus on the relative microbial contributions and lack microscopic and physicochemical explanations of the mechanisms promoting carbon sequestration, it is crucial to (a) verify the carbon sequestration capacity of A-HA-mediated Fe-C coupling; (b) investigate the spatial heterogeneity and redox sensitivity of A-HA/iron oxides that regulate the dynamics of OC accumulation; and (c) investigate the role of A-HA/Fe (hydr)oxide as the primary cementing agents for forming soil aggregates and achieving physical protection of SOC. We believe that such studies will enhance our understanding of the dynamic coupling processes between A-HA (as a defined model of soil organic matter) and iron ions, thereby promoting healthy biogeochemical cycles.

## Results

### Carbon retention capacity of A-HA-Fe coupling

**Fig. 1.**
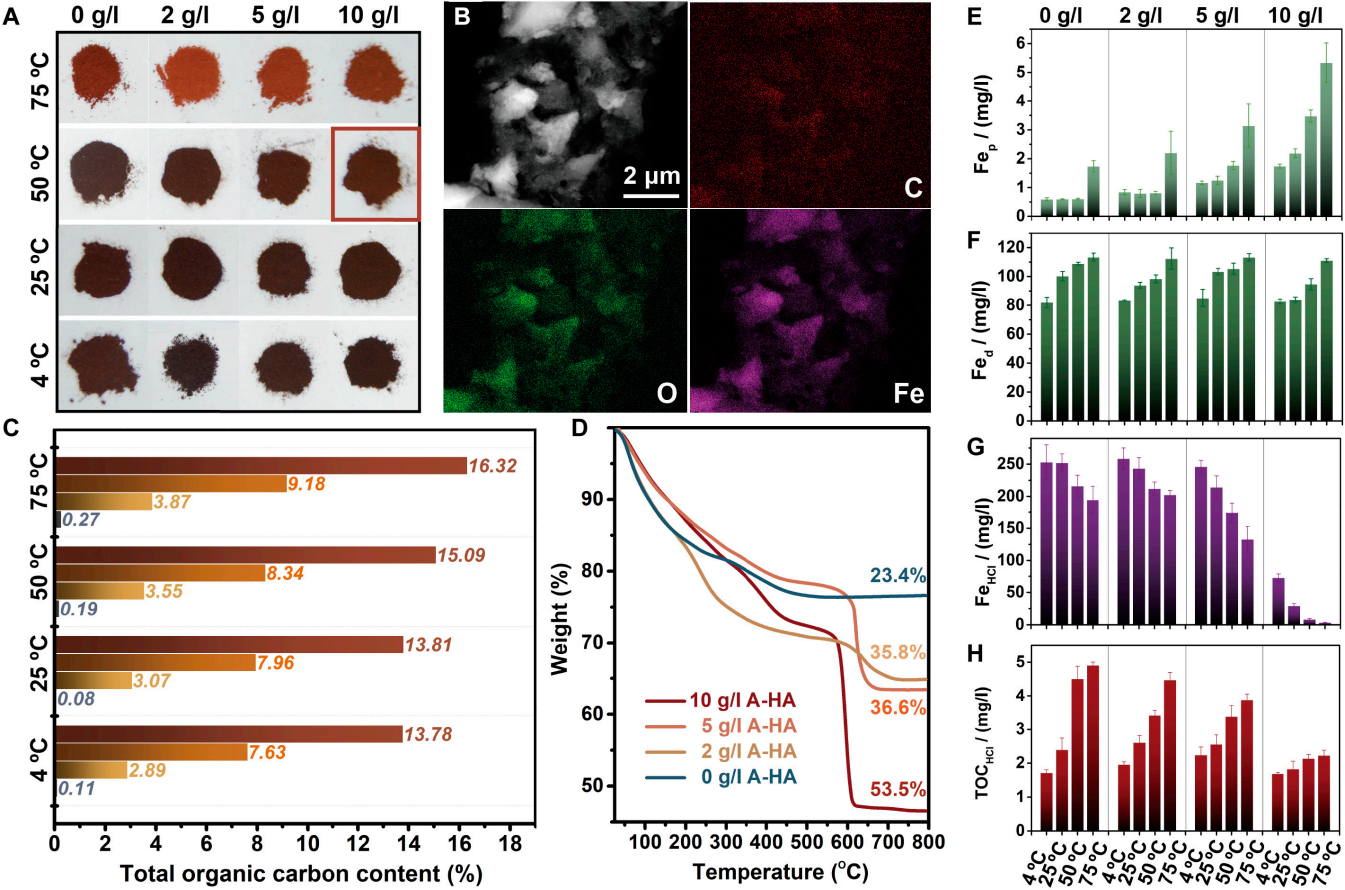
The capacity of Fe (hydr)oxide as a carbon (A-HA) sink. (A) The morphological colors of A-HA_0-10_/Fe (hydr)oxide under aging conditions with 4 to 75 °C. (B) Transmission electron microscopy (TEM)-mapping images of A-HA_10_/Fe (hydr)oxide-50. (C) C percentage of A-HA_0-10_/Fe (hydr)oxide under aging conditions with 4 to 75 °C. (D) Thermogravimetric spectra of A-HA_0-10_/Fe (hydr)oxide under aging conditions with 25 °C. (E) Organically complexed Fe oxides (Fe_p_), (F) total reactive Fe oxides (Fe_d_), (G) soil microbially available iron (Fe_HCl_), and (H) soil microbially available total organic carbon (TOC_HCl_) of A-HA_0-10_/Fe (hydr)oxide under aging conditions with 4 to 75 °C.

Figure 1A shows the variation of the coupling substances of OC (here referred to as A-HA) and dissolved Fe (hydr)oxides at different A-HA concentrations and temperatures. The variations in the colors of Fe (hydr)oxides are considered to reflect the interfacial reaction between A-HA and iron, even as a basis for a preliminary assessment of the mineral phases by visual observation (Munsell Color Classification System, Tables S1 and S2), such as yellow for goethite, blood red for hematite, and reddish-brown for ferrihydrite. The presence of Fe, O, and C elements in Fe (hydr)oxides further validates the similarity with natural OC–mineral dynamic processes (Fig. 1B). The Fe and C elements are uniformly distributed, as well as present with substantial overlap [19]. As depicted in Fig. 1C, the C content in Fe (hydr)oxides without A-HA ranged from 0.08% to 0.27%. After adding A-HA, C content increased to 2.89%–3.87% (2 g/l A-HA), 7.63%–9.18% (5 g/l A-HA), and 13.78%–16.32% (10 g/l A-HA). This suggests that more A-HA can be sequestered by Fe (hydr)oxides with higher concentrations of A-HA, providing direct mineral protection through adsorption, complexation, coprecipitation, and intercalation complex formation. Increasing temperatures also positively affected carbon fixation in neutral reaction systems, which is necessary to enhance the activity and rate of the reaction system [20].

Thermogravimetric mass loss was noted over the range of temperatures corresponding to the initial A-HA concentration in the adsorption and coprecipitation system (Fig. 1D). Further differential thermogravimetric and differential thermal analyses were conducted, with each peak representing the mass loss of a specific molecule or component (Fig. S1) [21]. Thermogravimetric data shows that the total weight loss visibly increases with the increase of coprecipitated A-HA concentrations (0, 2, 5, and 10 g/l) between 550 and 650 °C, ranging from 23.4% to 35.8%, 36.6%, and 53.5%, which was attributed to decomposition of recalcitrant carbon, such as aromatic compounds of lignin or polyphenols or polycondensed aromatic carbons [22]. Besides, differential thermogravimetric peak maxima are slightly shifted to lower temperatures (Fig. S1). In accordance with the research from Sodano et al., lower temperatures is mainly attributed to the presence of the A-HA/Fe (hydr)oxide, although the catalytic effect of the Fe on the decarboxylation reaction cannot be excluded [23,24]. Coprecipitation of A-HA to Fe (hydr)oxide showed strong selective retention of aromatic components, and initial complexation of Fe ions by aromatic carboxylic moieties and precipitation as carbon-rich A-HA/Fe (hydr)oxide associations contributed to the total carbon retention, especially at higher A-HA solution [23].

As shown in Fig. 1E, the addition of A-HA results in a considerable increase of Fe_p_ (organically complexed Fe oxides) contents in A-HA/Fe (hydr)oxide of 0.841 ± 0.094 to 2.203 ± 0.747, 1.167 ± 0.061 to 3.142 ± 0.754, and 1.743 ± 0.072 to 5.337 ± 0.687 mg/l with 2 to 10 g A-HA/l, as compared to that of Fe (hydr)oxide (0.591 ± 0.055 to 1.741 ± 0.195 mg/l). Meanwhile, there is no marked correlation between Fe_d_ (total reactive Fe oxides) and A-HA (Fig. 1F), supporting the hypothesis that A-HA/Fe (hydr)oxide composites can stabilize more “new carbon”. There are marked negative correlations of Fe_HCl_ and TOC_HCl_ (Fig. 1G and H), suggesting that the higher amounts of the A-HA associated OM and the precipitated insoluble Fe(III)−organic complexes are to be expected in which coprecipitation occurs at high C/Fe ratios. This is closely related to the chemically stabilized of Fe-bound A-HA functional groups, and precipitated insoluble A-HA/Fe complexes represent an apparently overlooked terrestrial carbon pool, and act as an “iron gate” and “carbon latch” in regulating OC dynamics [25–27].

### Organo–mineral nanometer interfaces of spatial heterogeneity in A-HA/Fe (hydr)oxide composition

**Fig. 2.**
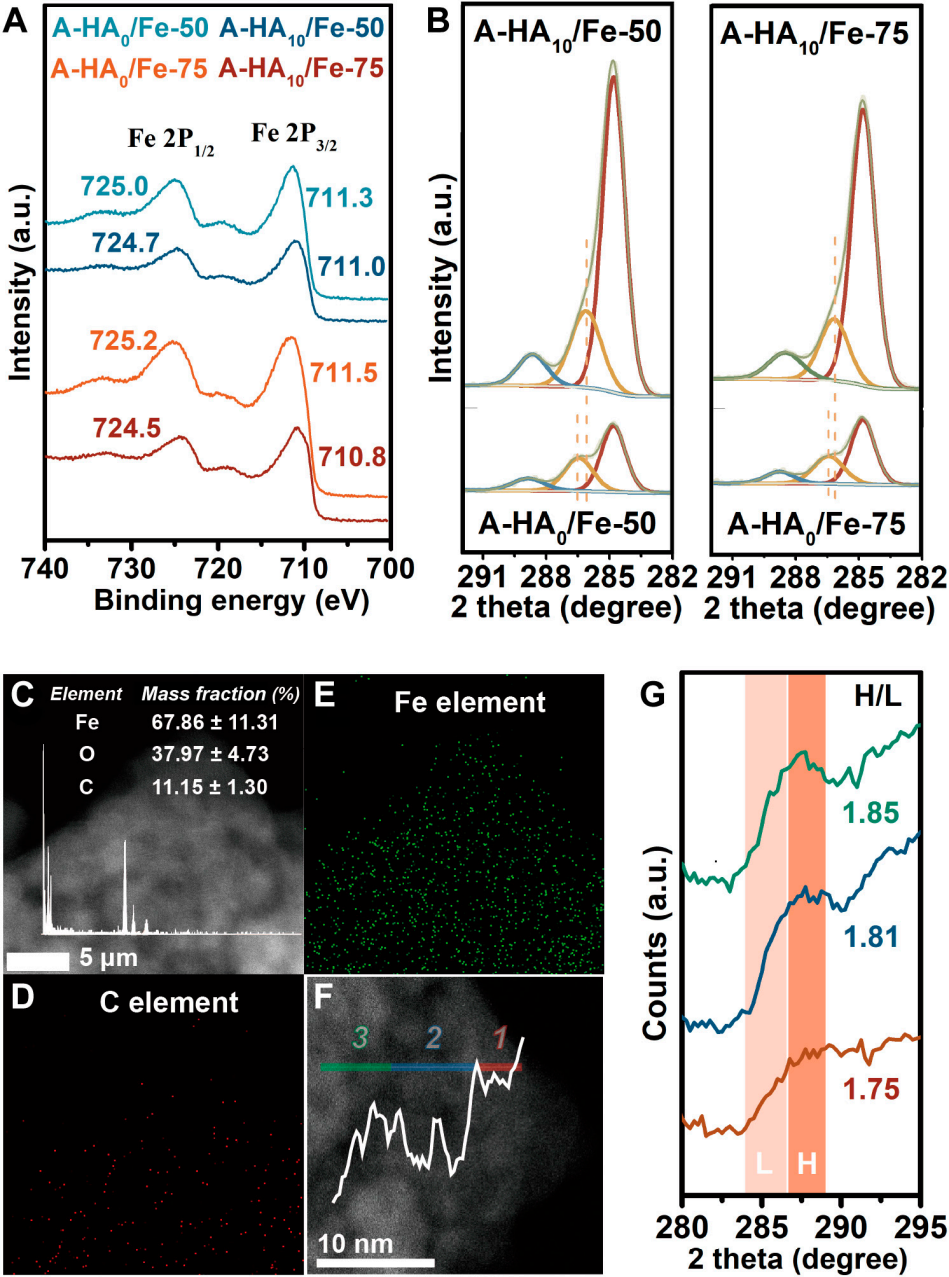
A-HA/Fe (hydr)oxide interfaces at the nanometer scale. (A) Fe2p and (B) O1s of XPS spectra in A-HA_0_/Fe (hydr)oxide-50/75 and A-HA_10_/Fe (hydr)oxide-50/75. (C to E) High-angle annular dark-field images and EDS elemental mapping of A-HA_10_/Fe (hydr)oxide-50. (F and G) STEM-EELS line scans of C of A-HA10/Fe (hydr)oxide-50. Three typical regions of C distribution are shown in the plots: the outer layer (red line, area 1), the intermediate layer (blue line, area 2), and the inner layer (green line, area 3). The EELS fine structure spectra of the 3 areas were collected.

Based on the above results, it can be predicted that A-HA may play an essential function in mediating carbon stability processes and promoting the formation of organo-metallic complexes, apart from acting as the carbon source. This supports the notion that Fe (hydr)oxides forms in solution and that organic ligands may be associated with iron (complexation) or present on the surface of nuclei or crystals. Thus, the binding energies of Fe2p1/2 and Fe2p3/2 in “organically complexed iron” was found to markedly decrease by 0.3 to 724.7/711.0 eV and by 0.7 to 724.5/710.8 eV under different aging crystallization conditions (pH = 7, *T* = 50 or 75 °C, and C_A-HA_ = 10 g/l) (Fig. 2A). The decreased binding energies could not be clearly explained by the electronegativity difference between A-HA and iron [28]. Instead, it was inferred that the Fe center of Fe (hydr)oxides undergoes a phase transition upon complexation and other reactions with A-HA [29]. X-ray diffraction (XRD) and Fourier transform infrared spectroscopy (FTIR) results confirmed that the introduction of A-HAs facilitated the crystallization of more stable Fe(III) crystalline forms such as goethite and hematite. In Figs. S2 and S3, 2 broad peaks of the XRD pattern appeared when the reaction temperature was adjusted to 50 °C. These peaks, combined with the characteristic peaks, indicate the presence of 2-line ferrihydrite. Upon the addition of a definite amount of 10 g/l A-HA, the main XRD diffraction is ascribed to hematite (PDF#98-000-0240), goethite (PDF#98-000-0229), and 2-line ferrihydrite. The FTIR absorption peaks are attributed to goethite bending vibrations and the hematite Fe-O absorption peaks. By analyzing fitted peak areas from x-ray photoelectron spectroscopy (XPS) data, the relative proportions of surface species were quantified. Deconvolution of the XPS C 1s profile shows the presence of carbon-related functional groups, such as C-C/C-H, aliphatic carbon (284.80 eV), O-C-O, aromatic C (286.11 to 286.66 eV), and O-C=O, carboxylic C (288.50 to 288.85 eV) (Fig. 2B and Table S3) [30,31]. The binding energies of the O-C-O and O-C=O groups in the A-HA/Fe coprecipitates shifted to lower values, and their proportions decreased markedly. This shift reveals the formation of precipitate and bridging complexes, which alters the electronic environment of the carbon atoms. Due to the greater electronegativity of hydrogen, fewer valence electrons of the carbon in the group are abstracted by iron after complexation, and the nuclear electrons experience a lower binding energy [32]. This result also suggests that aromatic C and carboxylic C groups interact with Fe (hydr)oxides through interfacial C-O-Fe bonds, which is in line with the FTIR results [31]. The increased or decreased intensity at ca. 1,716, 1,375 cm^−1^ provided evidence for the formation of complexes between A-HA and Fe (hydr)oxides (Fig. S3) [33,34].

Results of high-angle annular dark-field scanning transmission electron microscopy (STEM) surface scan at the nanoscale indicated that Fe, C, and O mass fraction in the complexing compound are (67.86 ± 11.31)%, (11.15 ± 1.30)%, and (37.97 ± 4.73)% (Fig. 2C). Substantially different spatial distribution patterns were observed for Fe and C elements (Fig. 2D and E), implying that the spatial heterogeneity of the Fe is associated with the C. In order to characterize the spatial heterogeneity and chemical features of organic C species at organo-mineral interfaces, EELS line scans of the spatial distribution of organic compounds near the surfaces of A-HA_10_/Fe-50 were performed as a function of distance [35] (Fig. 2F). The EELS line scan analysis suggests that the spatial distribution of C species is rather heterogeneous, with the C signal peaking close to the outer layer. In addition, similar to those in previous studies, distribution patterns of OC in the porous and sparse areas of Fe (hydr)oxides were also observed, which indirectly demonstrates the important role of nanopores or mineral interstitials in carbon sequestration [36,37]. To further investigate the spatial heterogeneity of oxidized and reduced carbon in A-HA_10_/Fe (hydr)oxide-50, we probed 3 specific areas to compare their H/L ratios [38]. Specifically, the A-HA_10_/Fe (hydr)oxide-50 interfacial region can be divided into 3 areas, the outer layer (red line, area 1), the intermediate layer (blue line, area 2), and the inner layer (green line, area 3), as denoted in Fig. 2F. The H/L ratios for area 2 and area 3 are similar, with H/L ratios ranging from 1.81 to 1.85, and slightly higher than that of area 3 (with an H/L ratio of 1.75). From the outer layer to the inner layer of the A-HA_10_/Fe (hydr)oxide-50 interface, the relative abundance of oxidized carbon increases and the relative abundance of reduced carbon decreases. This pattern suggests that carboxyl C is the most dominant organic C species in the inner layer, while aromatic C is mainly attached to the outer layer of the organic-mineral interface [35,38,39].

### Fe catalysis on the formation of OC geopolymerization-like A-HA/Fe (hydr)oxide

**Fig. 3.**
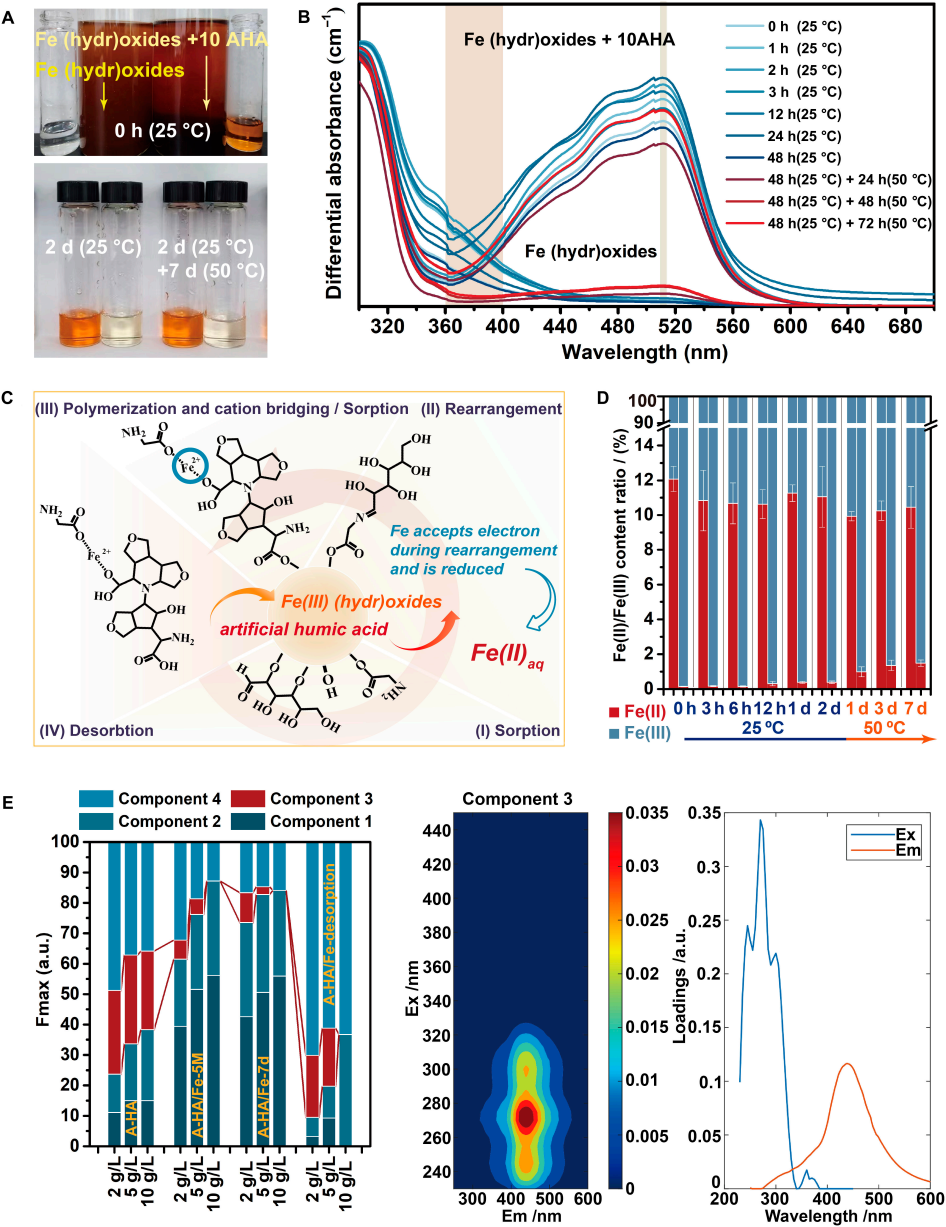
Fe catalysis of A-HA geopolymerization. (A) Color observation, (B) Ultraviolet-visible absorbance spectroscopy, (C) mechanism, and (D) Fe(II)/Fe(III) content ratio of reaction system with 1,10-phenanthroline in real time, sample data for each group, A-HA10/Fe (hydr)oxide-50 on the left, Fe (hydr)oxide-50 on the right. (E) EEM contours of fluorescent component 3 of 2 to 10 g/l A-HA, A-HA_2-5_/Fe (hydr)oxide-50 system reaction for 5 min supernatant (A-HA/Fe-5M), A-HA_2-5_/Fe (hydr)oxide-50 system reaction for 7 d supernatant (A-HA/Fe-7d), and Supernatant of 0.1 M NaH_2_PO_4_ desorbed A-HA_2-5_/Fe (hydr)oxide-50.

As shown in Fig. 3A and B, in a system where the color developer coexists with the A-HA/Fe (hydr)oxide complex, the processes of complex formation, rapid reduction of solid Fe to Fe^2+^ (510 nm), and maintenance of dynamic desorption can be observed in situ. The shape of the ultraviolet-visible absorption spectra increases linearly with wavelength in the range from 360 to 400 nm of A-HA/Fe (hydr)oxide precipitates (Fig. 3B). The emergence of these bands is postulated to be a result of modifications in interchromophore interactions and conformations within the A-HA molecules, which are caused by the accumulation of electric charge and/or other nonspecific effects related to deprotonation and Fe binding [40]. Moore et al. proposed that simple organic molecules can be geopolymerized (here refers to A-HA/Fe (hydr)oxide) into recalcitrant forms by the Maillard reaction [41]. As illustrated in Fig. 3C, we similarly attribute the catalytic effect of dissolved iron to a complexation mechanism to cation bridging. This creates a basis for the generation of more favorable free energy reactions. Firstly, the adsorption effect facilitates the clusters and orients of the reactants on the Fe (hydr)oxide surface, while the redox reaction between A-HA and the Fe (hydr)oxide generates dissolved Fe(II), which creates a bridging effect to form A-HA/Fe (hydr)oxide. Finally, dissolved Fe(II) may reoxidize and precipitate to A-HA/Fe (hydr)oxide for further catalytic reactions.

Figure 3E and Fig. S4 show the characteristic fluorescence spectra of the samples with the 4 fluorescent components identified by PARAFAC analysis. Among the transitions associated with fluorescence, quinones are characterized by excitation maxima in the range of 250 to 300 nm and distinct excitation peaks in the range of 330 to 400 nm due to the π→π* transitions of quinones and benzenes [42,43]. The excitation wavelength of the fluorescence component 3 of samples exactly matches that of the quinone component. Field and laboratory studies have shown that the quinone-like component is considered to be the most important redox-active functional group in humic substances, i.e. the electron chelating ability of A-HA/Fe (hydr)oxide is attributed to the quinone molecule of A-HA. The quinone fraction in (2 to 10) g/l A-HA accounted for 25.82% to 29.22% of the fluorescence of component 3. With the formation of the A-HA/Fe complex, the quinone fraction in the reaction solution decreases to (6.23 to 0)% and (9.82 to 0)% after 5 min and 7 d. The quinone-like components in the desorbed solution of A-HA/Fe complex accounted for 20.38% (10 g/l A-HA), 19.12% (5 g/l A-HA), and 0% (2 g/l A-HA) (2 g/l A-HA) of the fluorescence of component 3, respectively. That is, biomass humification provides a heterogeneous pool of quinones that produces “quinone boosting”, a high concentration of A-HA with a high quinone content and electron accepting capacity that achieves the reduction of Fe(III) to Fe(II). As shown in Fig. 3D, The Fe^2+^ content in A-HA/Fe (hydr)oxide was maintained at 12.076 ± 0.731 to 9.929 ± 0.263%, which was 84.250 to 6.595 times higher than the Fe^2+^ content in Fe (hydr)oxide. Our findings indicate that most of the reduced Fe remained in the solid phase as easily reoxidizable authigenic A-HA/Fe^2+^ (hydr)oxide. The solid-phase Fe^3+^/Fe^2+^ (hydr)oxide can serve as an immobile and rechargeable “redox battery”. The Fe “redox battery” is a characteristic feature of permeable environments or sediments with varying redox conditions, making Fe a key determinant of carbon turnover [44].

### Mediation from artificial humic acid on crystallization of Fe (hydr)oxides

**Fig. 4.**
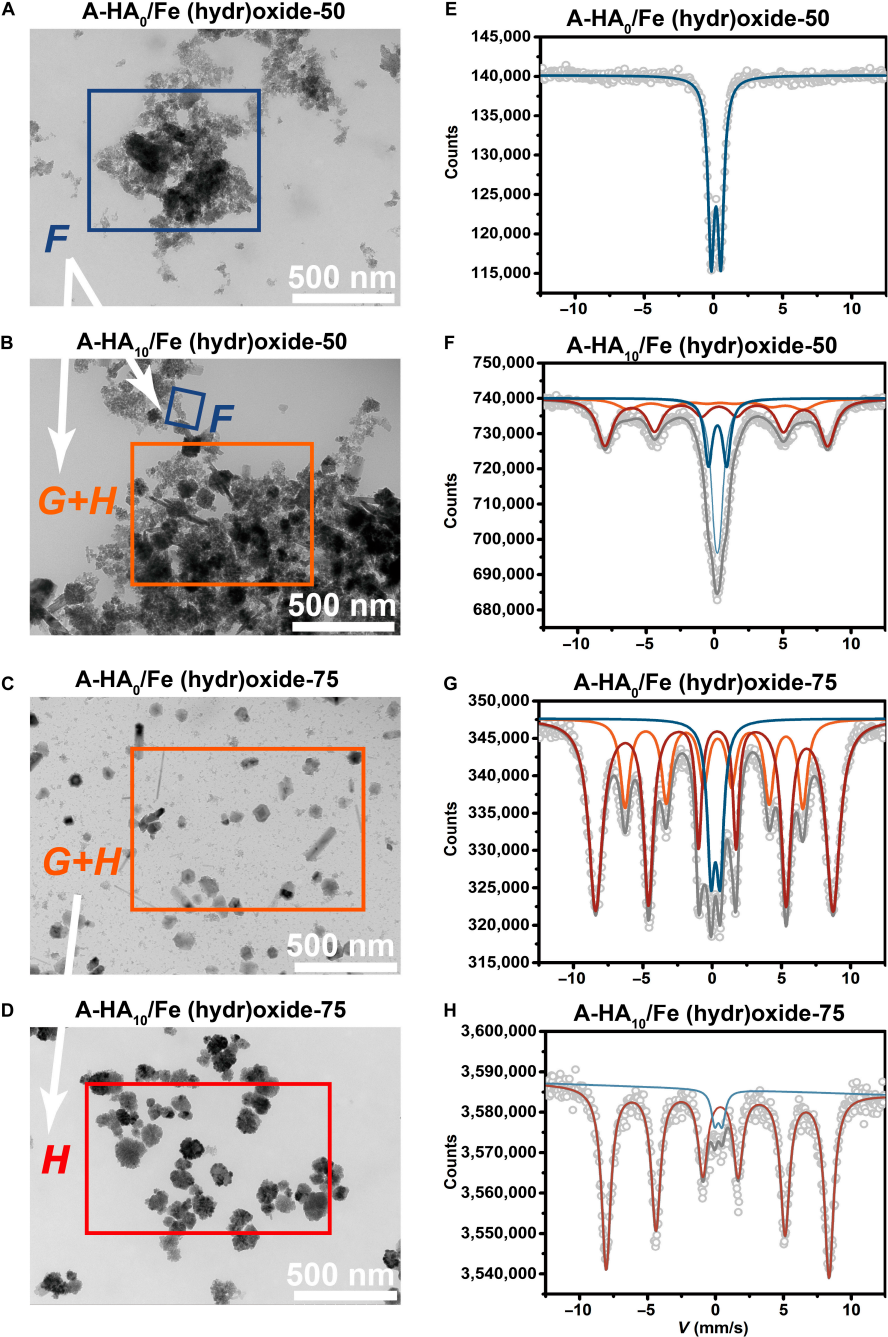
A-HA influence the crystallization processes of Fe (hydr)oxides. (A to D) TEM images and (E to H) room temperature Mössbauer spectra of A-HA/Fe (hydr)oxide.

The crystalline phases of A-HA/Fe (hydr)oxides incubated with neutral pH, 4 °C/25 °C/50 °C/75 °C temperatures, and varying A-HA concentrations (0 to 10 mg/l A-HA) were characterized with XRD patterns (Fig. S2). Notably, A-HA particularly influenced the crystallization of Fe (hydr)oxides. As shown in Fig. 4A and B, the initial ferrihydrite mainly appeared as dispersed primary particles (Fe (hydr)oxides system, *T* = 50 °C). After A-HA introduction, primary particles aggregated to form agglomerates, displaying needle-like, rounded clusters, and dispersed particles, which indicated the presence of oriented assembly (OA) in the crystallization process. A-HA induced A-HA/Fe(III) coprecipitates consisted of 2-line ferrihydrite, rounded cluster-like hematite, and needle-like goethite/hematite. When the aging temperature increased to 75 °C (Fig. 4C and D), coprecipitates even comprised pure hematite monomorph (50 to 100 nm). This demonstrates specificity of A-HA in allowing regulation of Fe (hydr)oxides. As mentioned earlier, the addition of A-HAs facilitated the crystallization of more stable Fe crystalline forms, such as goethite and hematite, which was accurately confirmed through room temperature Mössbauer data (Fig. 4E to H and Table S4) [45]. Mössbauer spectra allow us to characterize the crystallinity-continuum of Fe (hydr)oxides solid phases. The pathway of A-HA-regulated Fe (hydr)oxides was accurately determined, showing the crystallization progression from ferrihydrite (100%) to mixed phases of ferrihydrite (15.9%), goethite (14.3%), and hematite (44.4%), with a substantial increase in hematite fraction (60.7%) during crystallization, ultimately almost completely transitioning to hematite (94.7%). A-HA facilitated Fe (hydr)oxides transformation from superparamagnetic to magnetically ordered state [46]. The QS values of Fe (hydr)oxides varied with increasing C/Fe, indicating that A-HA affected the spatial coordination of central Fe^2+^/Fe^3+^ and octahedral structure around the central Fe atoms, which is essential for surface properties, morphology, and crystallization of Fe (hydr)oxides [47]. The Mössbauer spectra of the A-HA/ Fe (hydr)oxides yielded sextets, indicating electron transfer reactions occurred between the Fe^2+^ and native solution Fe^3+^ atoms during the 50 °C anoxic incubation [48]. Our results proved that coexisting A-HA/Fe (hydr)oxide precipitate promotes formation of more goethite or hematite via Fe(II)-precipitates electron transfer and template-directed nucleation and growth [49].

### Promotion of carbon sequestration in soil aggregates

**Fig. 5.**
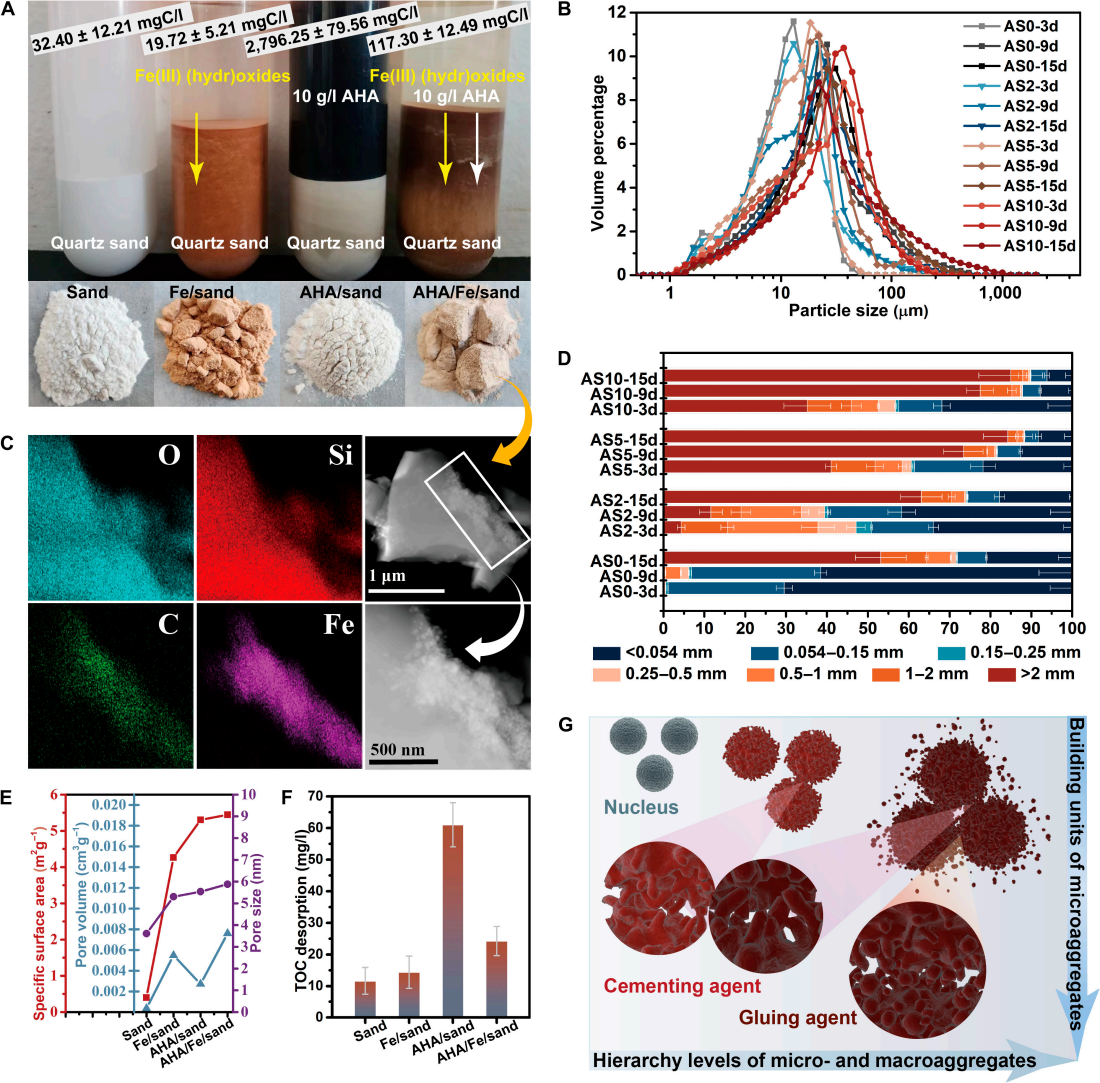
A-HA promotes soil aggregate formation for carbon sequestration. (A) Components and interaction effects of anthropogenic soil (AS). (B) Hierarchy of building units and microaggregates in anthropogenic soil. (C) TEM mapping of anthropogenic soil (A-HA/Fe/sand). (D) Particle size distribution determined by laser diffraction of AS with sand, A-HA, or Fe textures. (E) The N_2_ adsorption–desorption isotherm and BJH pore distribution of AS with sand, A-HA, or Fe textures. (F) Soil microbially available total organic carbon (TOC_HCl_) of AS with sand, A-HA, or Fe textures. (G) Size distribution and stability of anthropogenic soil aggregates.

The XRD result shows that, anthropogenic soil fraction was mainly SiO_2_ (Fig. S5), considering the low binding capacity of A-HA to quartz minerals (Fig. 5A). Across over long timescales, Fe (hydr)oxide can strongly affect on C levels through a variety of mechanisms, including adsorption of large amounts of dissolved OC (Fig. 5A), maintenance of OC stability, and decomposition and synthesis of complex OC [50]. Besides, colloidal Fe (hydr)oxide or A-HA-Fe (hydr)oxide associations is important building unit of the aggregates system, actively promoting the formation and stabilization of soil aggregates (Fig. 5B) [7].

As shown in Fig. 5C, Fe (hydr)oxide acts as a flocculant for the quartz sand and A-HA, and the microstructure (A-HA/Fe (hydr)oxide) is precipitated on the surface of the quartz sand as a “binder”, acting as “bridges”. This suggests that the microstructure-induced aggregate formation can connect particles of all sizes and underlines that also sand grains can be found within aggregates, contributing to larger aggregate sizes [51]. The cementing by A-HA/Fe (hydr)oxide can increase the diameters of the aggregates and the microscopic pore structure by connecting and gluing more subunits (Fig. 5B, D, and E). Asano and Wagai [52] demonstrated that micron/submicron-sized aggregates are formed from “presumed building blocks” consisting of organomineral-metal as effective binding agents, at least in siliceous soils. After 3 to 15 d of anoxic incubation, the aggregate structure of sterilization soil in related to the distribution of different soil aggregate sizes was altered obviously by the addition of A-HA (Fig. 5G). For the formation of larger microaggregates, A-HA as a gluing agent and more Fe (hydr)oxides as cementing agents are more essential to glue together building units composed of smaller microaggregates [9]. The percentage of micro-aggregates (< 0.25 mm) was greatly reduced, which decreased from 28.38% (AS0-15d) in the control group to 25.94% (AS2-15d), 11.69% (AS5-15d), and 10.23% (AS10-15d). Correspondingly, the proportion of >2 mm was increased markedly from 53.16 ± 6.21% in the control group to 63.06 ± 5.07%, 84.28 ± 6.03%, and 84.96 ± 7.83% for the same treatments. As shown in Fig. 5F, in consistent with the generally proven fact that incorporation into organomineral complexes and occlusion within microaggregates play a critical role in OC stability against biodegradation [38,50,53].

## Discussion

Numerous literatures indicates that Fe polycations bind to organic matter resulting in the formation of Fe-OM, which stabilize freshly inputted exogenous OC. To achieve SOM sequestration, it requires a deeper understanding of the abiotic and biotic mechanisms underlying carbon processing in soils. Especially, for iron-oxide-rich rice paddies or similar environments account for about 40% of the SOM sequestration potential in China [54]. This study, FT-IR, XPS, and Mössbauer spectra exhibited that A-HA influenced the spatial coordination of central Fe and the octahedral structure around central Fe atoms, with some of its O-C-O/O-C=O groups complexing with Fe to form coprecipitation complexes. Spherical aberration-corrected scanning transmission electron microscopy (Cs-STEM) coupled with electron energy loss spectroscopy (EELS) was used to analyze the microscopic surface behavior of A-HA with Fe and the transformation of organically complexed iron hydrolysis to more stable iron mineral phases were investigated under varying temperature conditions. This revealed the mineral preservation mechanism and the spatial heterogeneity of A-HA at the nanoscale, and highlighted the importance of local A-HA enrichment in controlling the chemical composition of OC. Furthermore, in consistent with numerous previous studies on the mineralogical characterization, the inner-sphere ligand exchanges of oxidized carboxylic and reduced aromatic acids of A-HA/Fe have been well documented and are proposed to be the key carbon forms for stabilizing interactions with Fe (hydr)oxide. The results of this study deliver a direct perspective on the species distribution of A-HA, supporting a layer-by-layer “onion” model of A-HA/Fe (hydr)oxide interface [37]. The micro-distribution of organic C species in A-HA/Fe (hydr)oxides at the nano- and sub-nanometre scales showed an increase in the relative abundance of oxidized carbon and a decrease in the relative abundance of reduced carbon from the outer to the inner layers of the interface. This pattern suggests that carboxylated carbon is the dominant OC species in the inner layer, while aromatic carbon is mainly attached to the outer layer at the organic-mineral interface.

This study focuses on the abiotic mechanisms of coupled Fe-C regulation for carbon sequestration, mainly involving complexation coprecipitation and soil aggregates formation. In the redox-active A-HA-Fe solution system, electron-rich (i.e., reduced) A-HA (HumS) abiotically reduces Fe^3+^ to Fe^2+^ [55]. When Fe^3+^ is reduced, the A-HA attached to the surface of Fe (hydr)oxides will decompose. The Fe^3+^/Fe^2+^ in the solid-state function as a rather stationary and rechargeable “redox battery”. Repeated electron transfers catalyzed reductive dissolution and subsequent exposure to aqueous ferrous (Fe^2+^) solutions and oxidative reprecipitation promote increased crystallinity and reactivity of the iron phase [49,56]. Stabilized iron phases with high crystallinity may prevent access to microorganisms and secreted enzymes, reducing the diffusion of oxygen to organic mineral microaggregates and contributing to the long-term retention, persistence, and accumulation of humic acids within soil minerals [57]. Besides, organic matter can contribute to the preservation of SOC by improving soil aggregation [58]. Microaggregates are formed through the interaction of polyvalent iron cations and A-HA ligands with the Fe (hydr)oxides surface, either through adsorption interactions or through coprecipitation. Fe (hydr)oxide acts as a flocculant for the quartz sand and A-HA, and the microstructure (A-HA/Fe (hydr)oxide) is precipitated on the surface of the quartz sand as a “binder”, acting as “bridges”. The formation of quartz sand–Fe (hydr)oxides–A-HA, is the main cementing mechanism for the generation of soil microaggregates. We propose that A-HA inputs could exert a substantial effect on the geochemistry of iron. A-HA-mediated Fe-C coupling has potential for carbon sequestration. Overall, the stabilization of OC is controlled by 3 main mechanisms: (a) chemically innate recalcitrance, (b) protection through interaction with Fe (hydr)oxide, and (c) occlusion in aggregates.

## Materials and Methods

### Materials

All chemicals used in this study were of ACS-grade quality. High purity water (with resistivity > 18 MΩ·cm) from a Millipore Milli-Q purification system was used to prepare solutions. Iron nitrate nonahydrate (Fe(NO_3_)_3_·9H_2_O), hydroxylamine hydrochloride, sodium polyphosphate decahydrate, ammonium oxalate, dithionite, ammonium fluoride, 1,10-phenanthroline, acetic acid, sodium acetate, potassium hydroxide (KOH), and hydrochloric acid (HCl) were acquired from Sinopharm Chemical Reagent Co., Ltd. The pure sand particles were quartz with a particle size of <0.0450 mm was obtained from Tianjin Damao Chemical Reagent Co., Ltd.

### Preparation of A-HA, A-HA/Fe (hydr)oxides complexes, and anthropogenic soil

A-HA was prepared using the hydrothermal humification method from our previous studies [59,60], with details provided in Text S1. Then, 1 g of A-HA was dissolved in 1 l of 6% ammonia solution to create a 10 g/l A-HA stock solution, which was later diluted to the desired concentration using high-purity water. Fe (hydr)oxides were synthesized based on the modified process from Schwertmann and Cornell [61,62], and the synthesis was repeated using various A-HA concentrations and different aging conditions. Specifically, 2 g of Fe(NO_3_)_3_·9H_2_O were dissolved in 20 ml of high purity water, and the pH was adjusted to approximately 8 using 0.1 to 1 mol l^−1^ NaOH or HCl to form flocculent substances. Then, 14 ml of 0, 2, 5, and 10 g/l A-HA solution was added, and the pH was further adjusted to 7 ± 0.05. The mixtures were aged for 7 d in water baths set at 4 ± 0.5, 25 ± 0.5, 50 ± 0.5, and 75 ± 0.5 °C, after vigorous shaking, abbreviated as A-HA_X_/Fe (hydr)oxide-Y (X = 0 to 10 g/l A-HA, Y = 4 to 75 °C). The temperature settings were obtained from Chen and Das et al. to increase the reaction rate of the system, validate experimental results over a wide temperature range, and reduce the interference of thermal transformations in the system [20,63]. The detailed experimental design can be found in Table S1. Anthropogenic soil (AS) was prepared by mixing 20 g the pure sand with the prepared A-HA/Fe (hydr)oxides, abbreviated as AS_X_ –Y (X = 0 to 10 g/l A-HA, Y = 3 to 15 d anthropogenic maturation). It should be noted that the construction of anthropogenic soil was carried out under aseptic conditions. Both the ferric ion and A-HA reaction systems were protected by nitrogen. Samples were sterilized in autoclaves before sampling and subsequent testing.

### Characterizations and quantitative analysis

TOC was assessed using a TOC analyzer (TOC-L CPN). The mineralogy of Fe (hydr)oxides and A-HA/Fe (hydr)oxides coprecipitates phase was characterized by XRD (Miniflex, Rigaku), and peak identification was performed using Jade 9.0 software. The molecular structures of Fe (hydr)oxides were analyzed by FTIR (Nicolet 460, Thermo Fisher). Particle morphology and size were characterized by TEM (HT7800, HITACHI). XPS (Escalab 250Xi, Thermo Fisher) was applied to measure the chemical composition on sample surfaces, and the data were analyzed using XPSPEAK software. The thermal stability of Fe (hydr)oxides was investigated through TGA (TGA8000, PerkinElmer) under a nitrogen atmosphere, heating from 25 to 800 °C at a rate of 20 °C/min. The ^57^Fe Mössbauer spectra of Fe (hydr)oxide was recorded on an SEE Co W304 Mössbauer spectrometer, using a ^57^Co/Rh source in transmission geometry. A fluorescence spectrometer (Shimadzu F-7000, Hitachi) was employed with a 150-W xenon lamp for the acquisition of EEM fluorescence spectrum. Direct visualization of the interfacial behavior between A-HA and Fe (hydr)oxide at the (sub)nanoscale using an advanced Cs-STEM [38]. Particle size distribution was obtained using the laser diffraction method on the Mastersizer 2000 with the Hydro G attachment. The quantitative analyses methods for Fe(III) and Fe(II) were referred to Sun, Jeewani, and Liao et al [50,54,64]. Detailed procedures for these instrumentation methods can be found in the Text S2. Real-time measurement of Fe(II) was performed using the modified 1,10-phenanthroline method. Fluoride was added to remove interference from Fe(III) in aqueous solution. The color developer was added to a closed nitrogen-filled reaction vessel of A-HA/Fe. A blank sample, consisting of the color developer and the reaction solution without A-HA, was used to reduce error.

### Anthropogenic soil aggregates stability tests

To determine the effects of A-HA/Fe (hydr)oxides on aggregates stability, ultrapure water was used to rapidly wet the AS aggregates. The following steps were taken: 20 g of AS samples were gently immersed in ultrapure water for 15 min, and excess liquid was then removed. The completely moistened AS was then transferred to sieves with pore sizes of 0.054, 0.15, 0.25, 0.5, 1, and 2 mm to separate aggregate fragments through wet sieving. The resistant soils on each sieve were recovered, dried at 60 °C, and weighed to obtain aggregate fractions of <0.054 mm, 0.054 to 0.15 mm, 0.15 to 0.55 mm, 0.25 to 0.5 mm, 0.5 to 1 mm, 1 to 2 mm, and >2 mm. All batches were performed in triplicate for further data analysis (unless otherwise indicated).

## Data Availability

The data that support the findings of this study are available within this paper and/or included in the Supplementary Materials and from the corresponding author upon request.
