# Supplementary material for: Artificial Humic Acid Mediated Carbon–Iron Coupling to Promote Carbon Sequestration
Source: Research (Wash D C). 2024 Feb 19;7:0308. doi: 10.34133/research.0308 (PMC10875824; doi:10.34133/research.0308)
Supplement: Supplementary 1 — Supplementary Text S1 to S3 Tables S1 to S5 Figs. S1 to S5 [file research.0308.f1.zip › Supplementary Materials.docx]

**Artificial Humic Acid Mediated Carbon - Iron Coupling to promote Carbon Sequestration**

Yibo Lan^a,b^, Shuang Gai^a,b^, Kui Cheng^b,c*^, Zhuqing Liu^a,b*^, Markus Antonietti^d^, Fan Yang^a,b*^

*^a^* School of Water Conservancy and Civil Engineering, Northeast Agricultural University, Harbin 150030.

*^b^* International Cooperation Joint Laboratory of Health in Cold Region Black Soil Habitat of the Ministry of Education, Harbin 150030.

*^c^* College of Engineering, Northeast Agricultural University, Harbin 150030.

*^d^* Max Planck Institute of Colloids and Interfaces Department of Colloid Chemistry 14476 Potsdam.

* Corresponding author: yangfan_neau@163.com

* Corresponding author: chengkui@neau.edu.cn

* Corresponding author: lzq@cau.edu.cn

**Text S1.** Preparation of artificial humic acid through hydrothermal humification process.

Weighed 24 g of eucalyptus leaf powders with a particle size of 100 mesh, added a certain amount of KOH (the mass ration of biomass and KOH is determined by composition of biomass) and 220 mL ultra-pure water to the autoclave in oven with 200 °C for 24 h, and then cooled down by water with subsequent filtration to obtain liquid products. At last, adjusted the pH of liquid products to 1 using HCl (2.0 mol/L) to obtain the A-HA solid, centrifuged A-HA solids to neutral with ultra-pure water and drying in oven with 80°C. The prepared A-HA sample was preserved for the next experiments and characterizations.

**Text S2.** Characterizations and quantitative analysis

**a. *Atomic absorption spectrometer:*** Total concentrations of Fe^3+^ in the Fe (hydr)oxides were analyzed by flame atomic absorption spectrometry after samples digestion according to the procedure provided by the manufacturer.

**b. *Total organic carbon (TOC)****:* The analysis is based on high-temperature catalytic combustion of the sample and subsequent detection of the released carbon dioxide. Sample analysis takes place in two stages: total carbon is determined by sample combustion at 900 °C; inorganic carbon, at 200 °C with the addition of phosphoric acid. Organic carbon is calculated by subtracting the content of inorganic carbon from the total carbon content.

**c. *X-ray diffraction (XRD)****:* XRD patterns were obtained using a copper target (Cu–Kα), a crystal graphite monochromator and a scintillation detector. The diffractometer was operated at a tube voltage of 40 kV and a tube current of 30 mA with step scanning from 2θ values of 10 to 90°, sequential increments of 0.02° and a scan speed of 2°/min.

**d.** ***Transmission electron microscopy (TEM)****:* The accelerating voltage was 200 kV. The particles were sonicated in ethanol for 3–5 min. Next, 1 or 2 drops of the solution were pipetted on a porous carbon film supported by a copper grid. As the remaining ethanol evaporated completely, the copper grids were placed in a vacuum chamber for analysis.

**e.** ***Fourier Transform Infrared (FTIR) spectroscopy****:* 1.0 mg of the dried sample was ground and mixed in an agate mortar at a mass ratio of 1:150 with dry KBr powder, and the measurement was performed at a wavelength of 400–4000 cm^−1^.

**f.** ***X-ray photoelectron spectroscopy (XPS)****:* XPS of the dried samples was performed by using an Al-Kα monochromatic X-ray source (1486.6 eV), and before determination, binding energy (B.E.) values were corrected to the carbon peak C 1s at 284.8 eV. The related acquisition parameters are as follows: the measured area was 650 μm, the lens mode was standard, the energy range was 0–1350 eV, and the energy step size was 1 eV.

**g.** ***Thermoanalyses (DTA-TGA)****:* The DTA-DTG analyses were done using a TGA8000 detector at a heating rate of 20ºC/min, the hold temperature was 800 ºC. The sample chamber was flushed with dry N_2_ during the run and the flow rate was 30 ml/min. A total mass of approximately 35 mg of finely ground sample was heated in an open platinum crucible.

**h.** ***Mössbauer spectroscopy****:* Mössbauer measurements were performed in transmission mode using a constant-acceleration spectrometer. ^57^Fe resonant absorption spectra were obtained on 25 mg of sample powder that is packed into a pre-drilled hole of desired dimension in a Pb disc in order to obtain a sample thickness of ~5 mg Fe/cm^2^. A Ta foil with a hole of appropriate diameter was then mounted on the Pb disc to ensure that only gamma rays that had interacted with the sample reached the detector. The spectrometer was operated with a ^57^Co/Rh source in rhodium that was calibrated using α-Fe. The spectra were collected over a velocity range of approximately ±5 mm/s with a 512-channel multichannel analyzer. The velocity was calibrated with respect to a-Fe metal at room temperature. The raw data were folded to obtain a flat background. Data were fitted using the MossWinn 4.0 software.

**i. *Excitation-emission matrix (EEM) fluorescence spectrum***: A fluorescence spectrometer equipped with water bath system was employed for the acquisition of EEM fluorescence spectrum with the ranges of excitation wavelength (Ex) and emission wavelength (Em), the scan increments of Ex and Em were 1 nm and 0.5 nm, with slits of 5 nm. Fluorescence measurement was conducted using a spectro-fluorometer () equipped at an ambient temperature of 25 °C. PARAFAC modeling was using the DOM Fluor toolbox in MATLAB 2015a. PARAFAC was computed using two to seven component models with non-negativity constraints, and then residual analysis, split-half analysis, and visual inspection were applied to determine the number of fluorescence components.

**j. *Spherical aberration corrected scanning transmission electron microscopy (Cs-STEM)****:* With the development of advanced Cs-STEM, direct visualization of C and other elements on A-HA/Fe (hydr)oxides at the (sub)nano scale has become possible with high angle annular dark field (HAADF) images, energy dispersive X-ray spectroscopy (EDS), and electron energy loss spectroscopy (EELS) elemental mapping

**k.** ***Particle size distribution****:* Particle size distribution was determined with the laser diffraction method on the Mastersizer 2000 with the Hydro G attachment (wet measurement), with the following sets: refractive index (RI) 1.52, measurement time 60 s, absorption index (AI) 0.1, stirrer speed 700 rpm, pump speed 1750 rpm.

**l. *Nitrogen adsorption–desorption isotherms***: Nitrogen adsorption–desorption isotherms of as-synthesized complexes were used to detect the surface area and porosity analyzer (ASAP 2020 HD88, Micromeritics).

**m.** With the development of advanced spherical aberration corrected scanning transmission electron microscopy (Cs-STEM), direct visualization of C and other elements on A-HA/Fe (hydr)oxides at the (sub)nano scale has become possible with high angle annular dark field (HAADF) images, energy dispersive X-ray spectroscopy (EDS), and electron energy loss spectroscopy (EELS) elemental mapping.

**n. *Quantitative analysis of Fe(III) and Fe(II)****:* To determine iron content, 2 mg of Fe(III) (hydr)oxides were dissolved in 2 mL of 37% HCl and subsequently diluted to an appropriate concentration using high purity water. Total Fe content was measured using an atomic absorption spectrometer (iCE3500, Thermo Fisher, USA). Organically complexed Fe oxides (Fe_p_) were extracted using sodium pyrophosphate and Dithionite-citrate-bicarbonate (DCB) extraction was used to quantify total reactive Fe oxides (Fe_d_). Soil micro-organisms can utilize this type of iron extracted by 0.5 M HCl, which mainly includes water-soluble iron, some amorphous iron, and complexed iron. The concentrations of aqueous Fe(II) (after filtration) and total Fe(II) (after digesting the unfiltered samples in 6 M HCl for 24 h) were using the modified 1,10-phenanthroline method with fluoride added to remove interference from aqueous Fe(III) at 510 nm on a UV–vis spectrophotometer (UV-2550, Shimadzu, Japan). Total dissolved iron was also analyzed by this method using hydroxylamine hydrochloride as the reducing agent . Total Fe concentration was assayed through reduction of Fe(III) to Fe(II) by 10% hydroxylamine hydrochloride. The procedure was carefully conducted in an anoxic cabinet to minimize the potential oxidation of Fe(II).

**Text S3.** The XRD data analyses

When the reaction temperature was adjusted to 50°C, two broad peaks of XRD pattern appeared at around 2θ=35° (110) and 2θ=62° (300), can demonstrate that the 2-line ferrihydrite was the only product. And with the addition of definite amount of 10 g/L A-HA, the main XRD diffraction peaks located at 2θ=24.150° (012), 33.162° (104), 35.630° (110), 40.863° (113), 49.464° (024), 54.074° (116), 57.608° (018), 62.437° (214), 63.999° (300), 71.962° (1010) are ascribed to hematite (PDF#98-000-0240). The other diffraction peaks presented at 2θ=21.240° (110), 34.686° (021), 36.658° (111), 58.997° (151) are identified as goethite (PDF#98-000-0229) and peaks near 2θ=35° (110) and 2θ=62° (300) are classified as 2-line ferrihydrite.

**Table S1.** Diagnostic reference of iron oxide minerals through munsell colour.

| **Mineral** | **Colour** | **Munsell** |
| --- | --- | --- |
| ***Ferrihydrite*** | Dark Reddish/Brown | 5YR - 7.5YR |
| ***Goethite*** | Brownish-Reddish/ Yellow | 7.5YR – 10YR |
| ***Hematite*** | Red | 5R - 2.5YR |

**Table S2.** Munsell Colours of synthetic Fe (hydr)oxides

| **Constant water bath temperature (T)** | **4****℃** | **10 g/L A-HA** 5YR 3/2 Dark Reddish Brown  **5 g/L A-HA** 5YR 3/2 Dark Reddish Brown  **2 g/L A-HA** 5YR 3/2 Dark Reddish Brown  **0 g/L A-HA** 5YR 3/2 Dark Reddish Brown |
| --- | --- | --- |
|  | **25℃** | **10 g/L A-HA** 5YR 3/2 Dark Reddish Brown  **5 g/L A-HA** 5YR 3/2 Dark Reddish Brown  **2 g/L A-HA** 5YR 3/2 Dark Reddish Brown  **0 g/L A-HA** 5YR 3/2 Dark Reddish Brown |
|  | **50℃** | **10 g/L A-HA** 5YR 3/3 Dark Reddish Brown  **5 g/L A-HA** 5YR 3/3 Dark Reddish Brown  **2 g/L A-HA** 5YR 3/2 Dark Reddish Brown  **0 g/L A-HA** 5YR 3/2 Dark Reddish Brown |
|  | **75℃** | **10 g/L A-HA** 2.5YR 4/4 Dull Reddish Brown  **5 g/L A-HA** 2.5YR 4/6 Reddish Brown  **2 g/L A-HA** 2.5YR 5/8 Bright Brown  **0 g/L A-HA** 2.5YR 3/4 Dark Reddish Brown |

**Table S3.** XPS C1s analysis results for Fe (hydr)oxides electrodes.

|  | **C-C**  **peak position and species ratio** | **O-C-O**  **peak position and species ratio** | **O-C=O**  **peak position and species ratio** |
| --- | --- | --- | --- |
| **A-HA_10_/Fe-50** | 284.80 eV  68.80% | 286.11 eV  22.40% | 288.66 eV  8.80% |
| **A-HA_0_/Fe-50** | 284.80 eV  54.40% | 286.45 eV  32.10% | 288.85 eV  13.50% |
| **A-HA_10_/Fe-75** | 284.80 eV  71.10% | 286.19 eV  19.16% | 288.50 eV  9.74% |
| **A-HA_0_/Fe-75** | 284.80 eV  57.18% | 286.42 eV  30.43% | 288.76 eV  12.39% |

**Table S4.** Fitted Mössbauer parameters for Fe (hydr)oxides. Isomer shift (IS), quadrupole splitting (QS), hyperfine field (H) values given with respect to Fe.

|  |  | **IS (mm/s)** | **QS (mm/s)** | **H (T)** | **Γ (mm/s)** | **Area (%)** | **Note** |
| --- | --- | --- | --- | --- | --- | --- | --- |
| A-HA_0_/Fe-50 | Doublet | 0.19 | 0.70 | — | 0.54 | 100 | Ferrihydrite |
| A-HA_10_/Fe-50 | Doublet | 0.22 | 1.34 | — | 0.69 | 15.9 | Ferrihydrite |
|  | Sextet1 | 0.27 | -0.37 | 39.88 | 2.0 | 14.3 | Goethite |
|  | Sextet2 | 0.23 | -0.20 | 50.64 | 1.34 | 44.4 | Hematite |
| A-HA_0_/Fe-75 | Doublet | 0.24 | 0.66 | — | 0.66 | 13.1 | Ferrihydrite |
|  | Sextet1 | 0.27 | -0.27 | 39.88 | 0.81 | 26.2 | Goethite |
|  | Sextet2 | 0.26 | -0.21 | 53.27 | 1.18 | 60.7 | Hematite |
| A-HA_10_/Fe-75 | Doublet | 0.21 | 0.54 | — | 0.58 | 5.3 | Ferrihydrite |
|  | Sextet2 | 0.26 | -0.21 | 50.98 | 0.82 | 94.7 | Hematite |

**Table S5.** The detailed experimental design.

|  | | **Adjusted solution pH of Fe (hydr)oxide (2g Fe(NO)_3_·9H_2_O + 20 mL H_2_O)** |
| --- | --- | --- |
| **Constant water bath temperature (T)** | **4℃** | + 14 mL A-HA (10 g/L) A-HA_10_/Fe-4 or A-HA_10_/ Fe (hydr)oxide-4 |
|  |  | + 14 mL A-HA (5 g/L) A-HA_5_/Fe-4 or A-HA_5_/ Fe (hydr)oxide-4 |
|  |  | + 14 mL A-HA (2 g/L) A-HA_2_/Fe-4 or A-HA_0_/ Fe (hydr)oxide-4 |
|  |  | + 14 mL H_2_O A-HA_0_/Fe-4 or A-HA_2_/ Fe (hydr)oxide-4 |
|  | **25℃** | + 14 mL A-HA (10 g/L) A-HA_10_/Fe-25 or A-HA_10_/ Fe (hydr)oxide-25 |
|  |  | + 14 mL A-HA (5 g/L) A-HA_5_/Fe-25 or A-HA_5_/ Fe (hydr)oxide-25 |
|  |  | + 14 mL A-HA (2 g/L) A-HA_2_/Fe-25 or A-HA_2_/ Fe (hydr)oxide-25 |
|  |  | + 14 mL H_2_O A-HA_0_/Fe-25 or A-HA_2_/ Fe (hydr)oxide-25 |
|  | **50℃** | + 14 mL A-HA (10 g/L) A-HA_10_/Fe-50 or A-HA_10_/ Fe (hydr)oxide-50 |
|  |  | + 14 mL A-HA (5 g/L) A-HA_5_/Fe-50 or A-HA_5_/ Fe (hydr)oxide-50 |
|  |  | + 14 mL A-HA (2 g/L) A-HA_2_/Fe-50 or A-HA_2_/ Fe (hydr)oxide-50 |
|  |  | + 14 mL H_2_O A-HA_0_/Fe-50 or A-HA_2_/ Fe (hydr)oxide-50 |
|  | **75℃** | + 14 mL A-HA (10 g/L) A-HA_10_/Fe-75 or A-HA_10_/ Fe (hydr)oxide-75 |
|  |  | + 14 mL A-HA (5 g/L) A-HA_5_/Fe-75 or A-HA_5_/ Fe (hydr)oxide-75 |
|  |  | + 14 mL A-HA (2 g/L) A-HA_2_/Fe-75 or A-HA_2_/ Fe (hydr)oxide-75 |
|  |  | + 14 mL H_2_O A-HA_0_/Fe-75 or A-HA_2_/ Fe (hydr)oxide-75 |


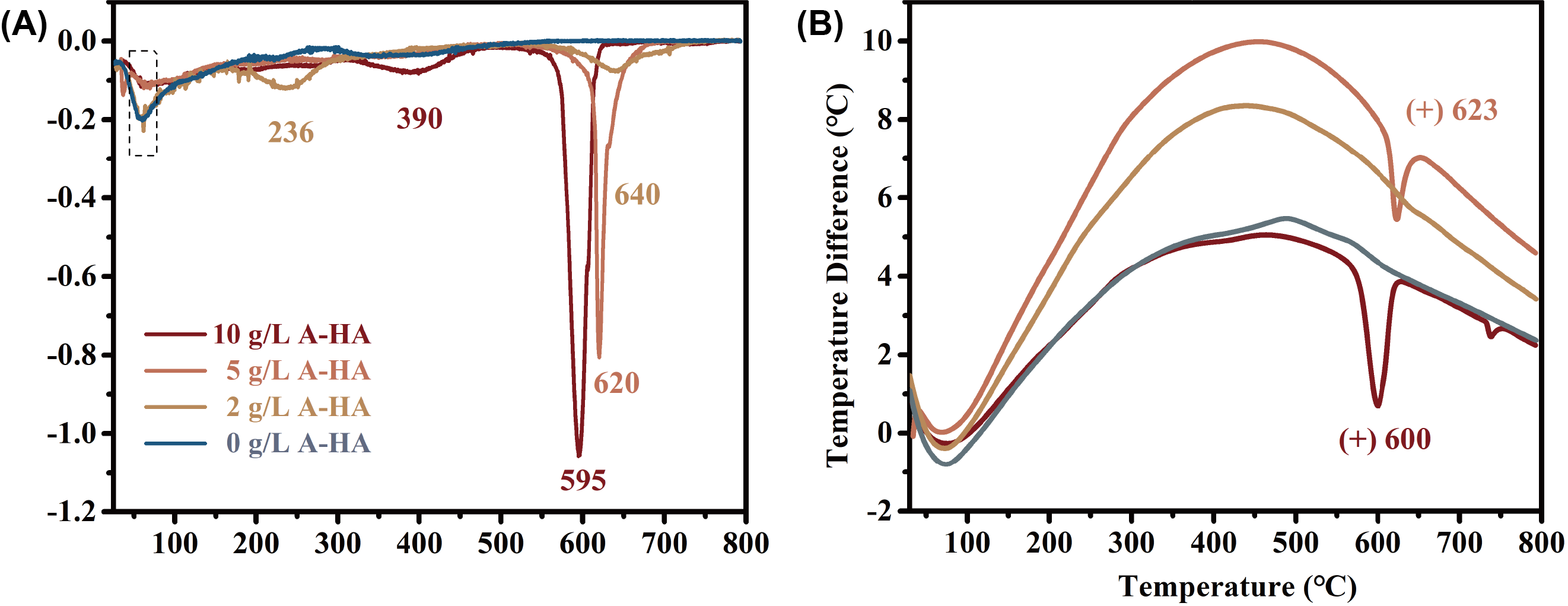


**Fig. S1.** (A) DTG and (B) DSC curves of Fe (hydr)oxides under aging conditions with 25ºC.


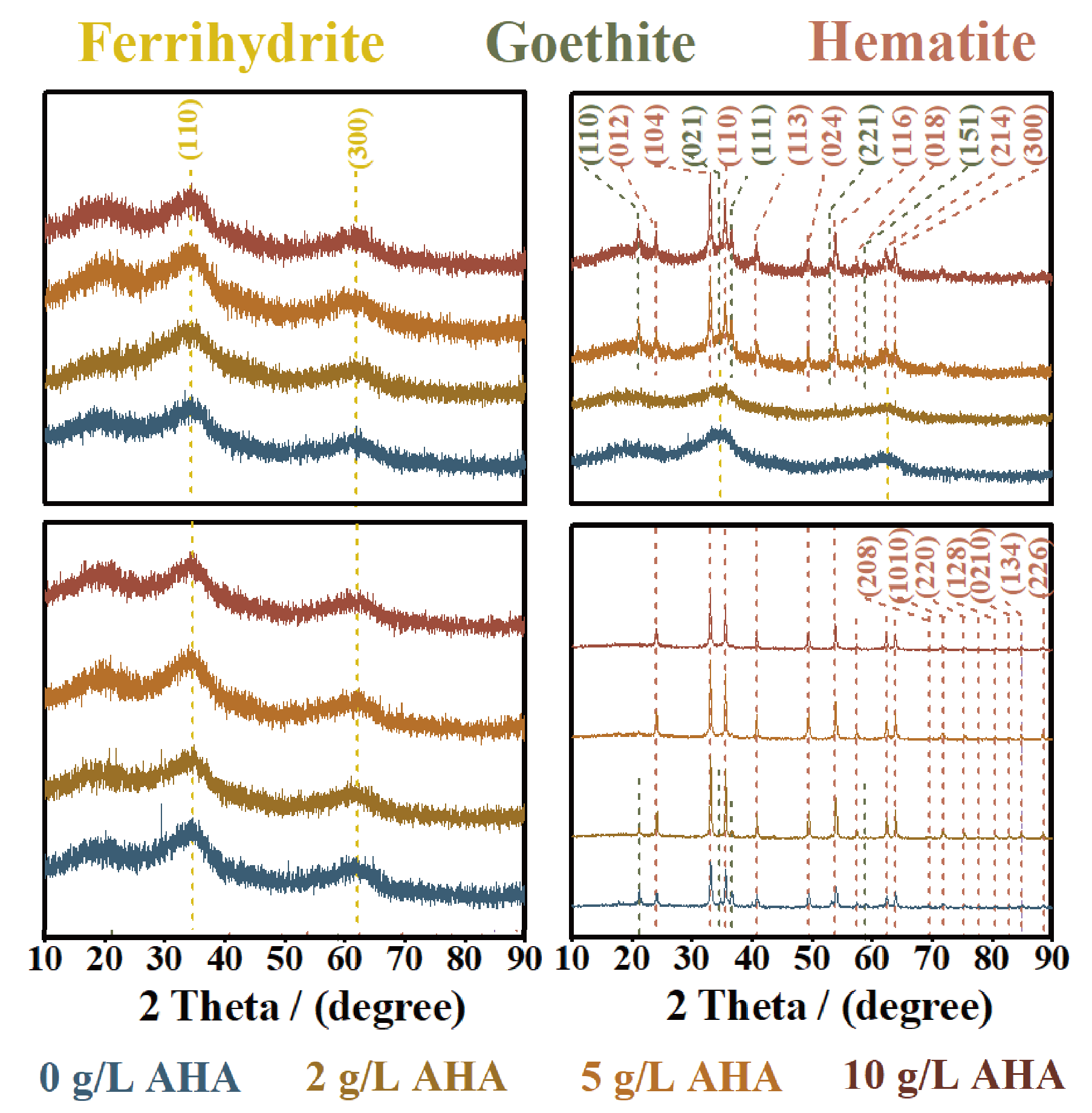


**Fig. S2.** XRD patterns of Fe (hydr)oxides under different A-HA concentrations (0, 2 g/L, 5 g/L and 10 g/L).


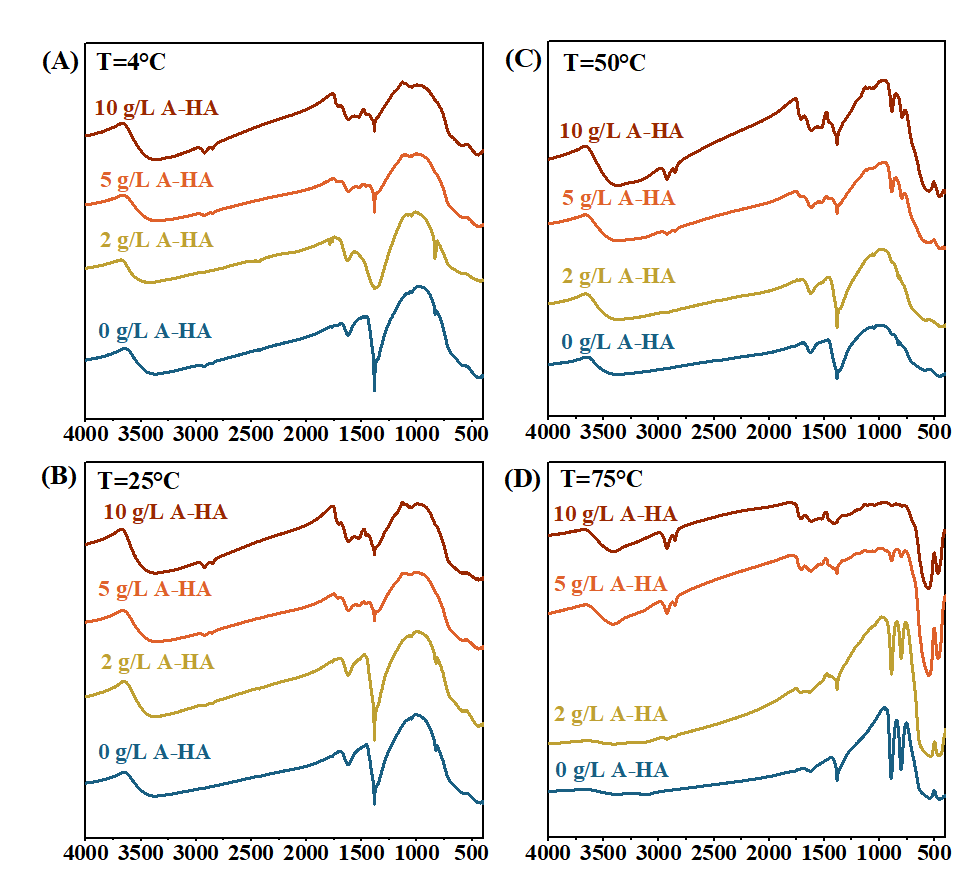


**Fig. S3.** FTIR patterns of Fe (hydr)oxides with different A-HA concentrations (0, 2 g/L, 5 g/L, and 10 g/L).


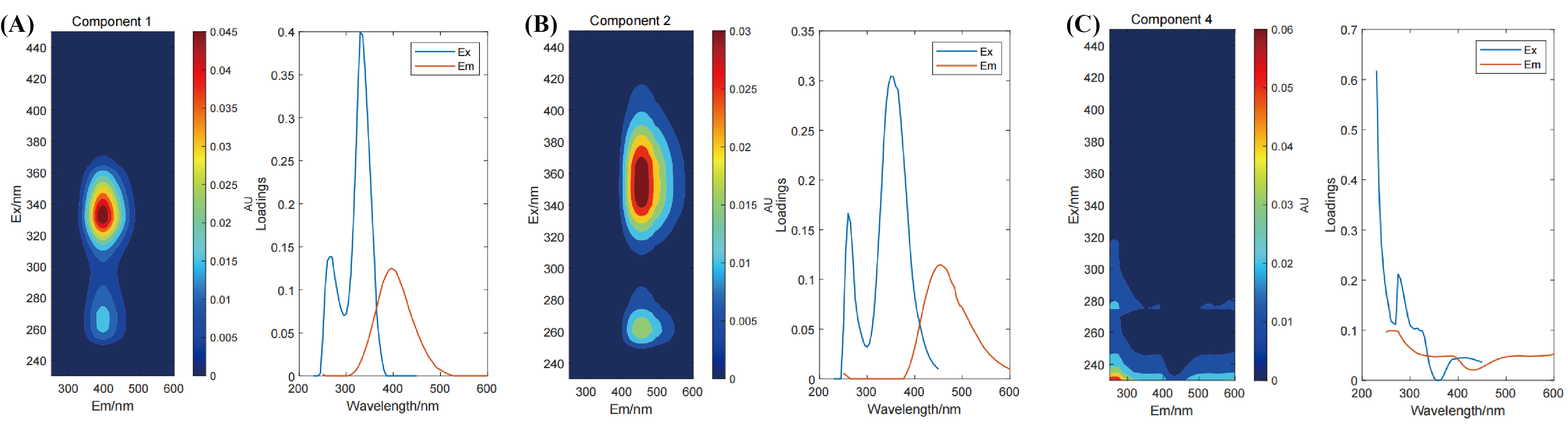


**Fig. S4.** EEM contours of five fluorescent component 1, component 2, and component 4.

\

**

**

**Fig. S5.** XRD patterns of AS with Sand, A-HA, or Fe textures.
