# Supplementary figures and images for: Artificial Humic Acid Mediated Carbon–Iron Coupling to Promote Carbon Sequestration
Source: Research (Wash D C). 2024 Feb 19;7:0308. doi: 10.34133/research.0308 (PMC10875824; doi:10.34133/research.0308)

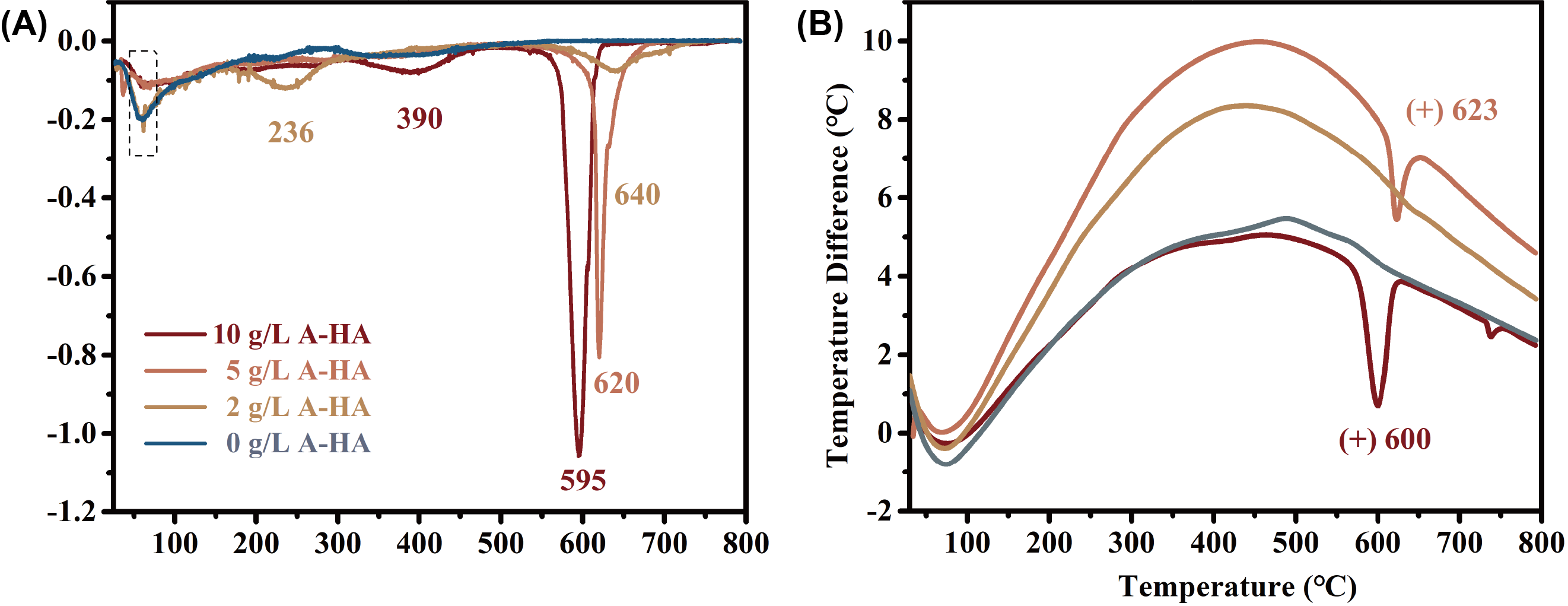

Supplement: Supplementary 1 — Supplementary Text S1 to S3 Tables S1 to S5 Figs. S1 to S5 [file research.0308.f1.zip › Fig.S1.png]

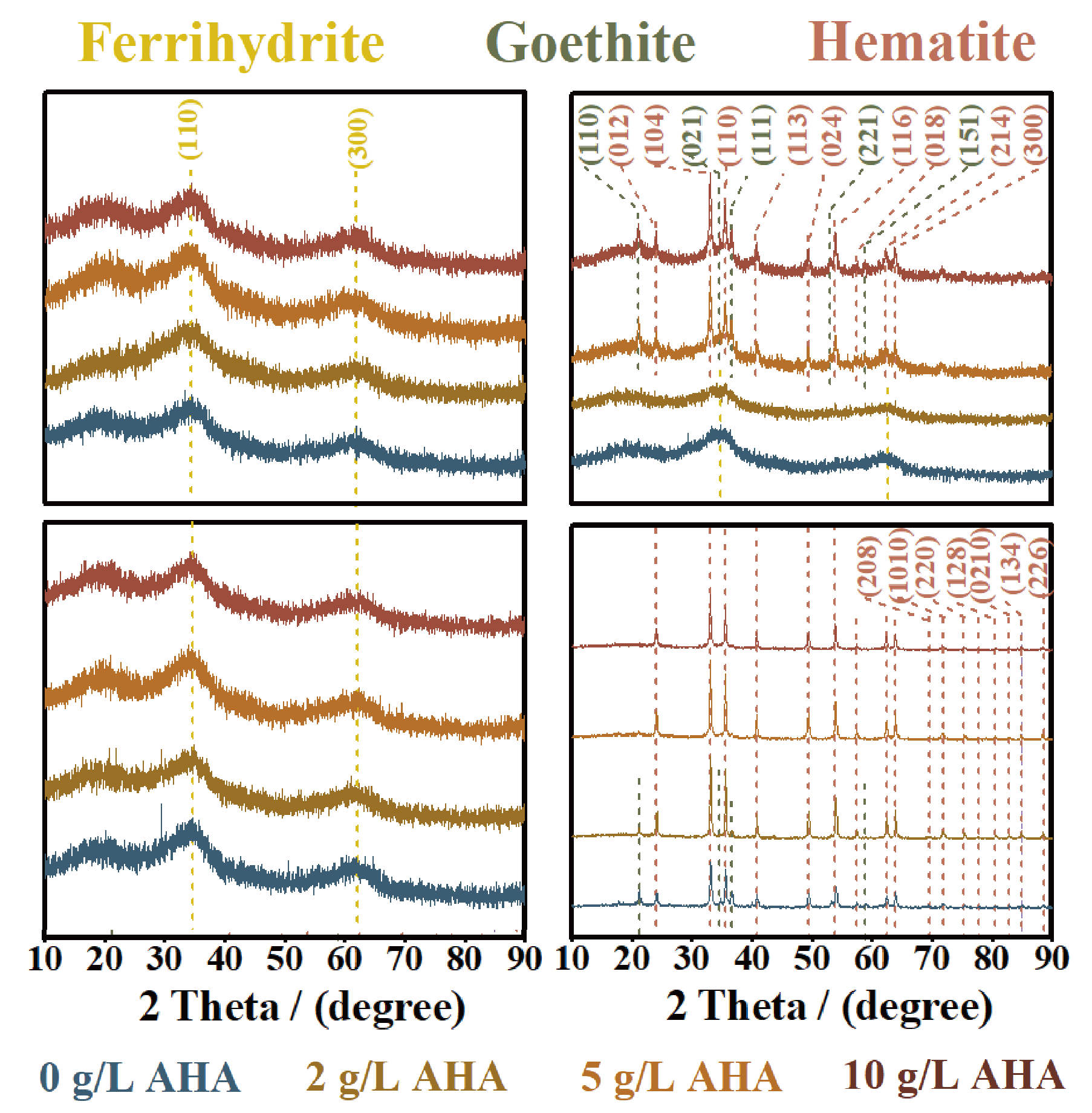

Supplement: Supplementary 1 — Supplementary Text S1 to S3 Tables S1 to S5 Figs. S1 to S5 [file research.0308.f1.zip › Figure S2.png]

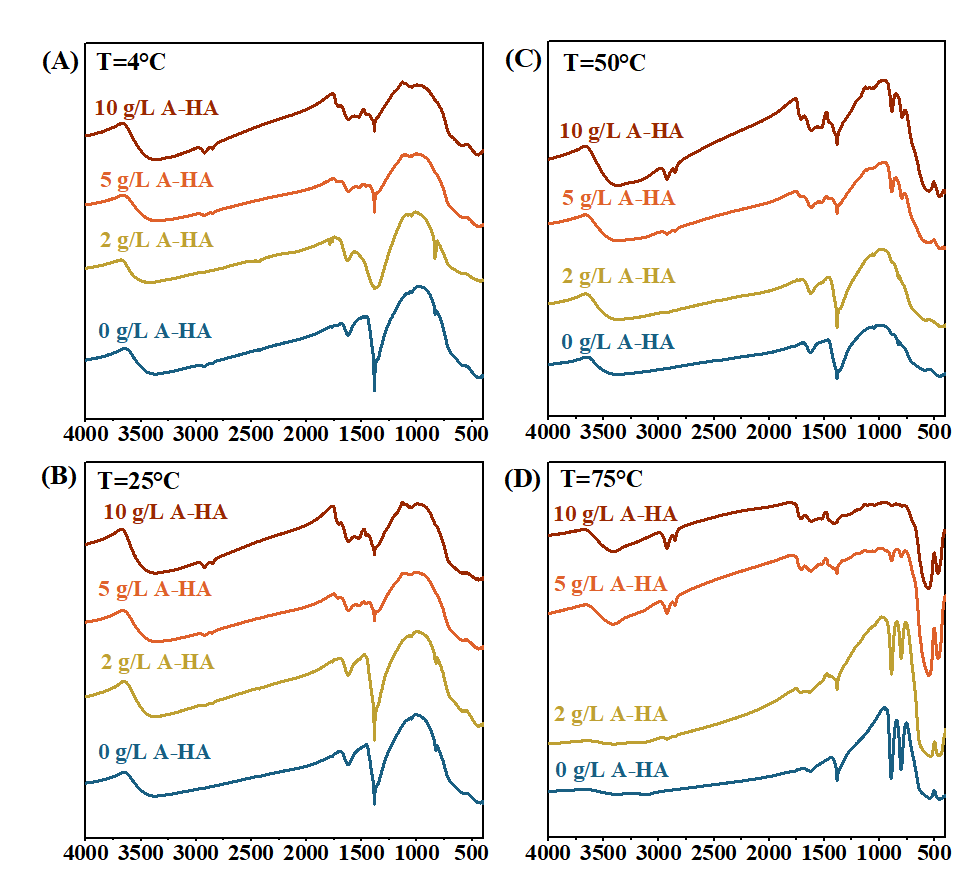

Supplement: Supplementary 1 — Supplementary Text S1 to S3 Tables S1 to S5 Figs. S1 to S5 [file research.0308.f1.zip › Figure S3.png]

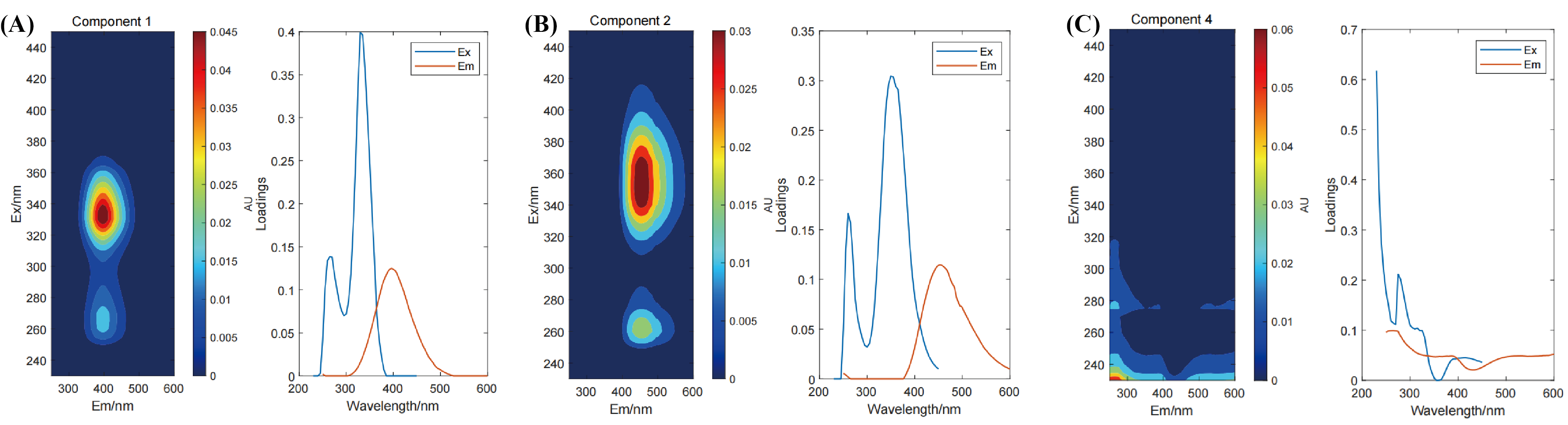

Supplement: Supplementary 1 — Supplementary Text S1 to S3 Tables S1 to S5 Figs. S1 to S5 [file research.0308.f1.zip › Figure S4.png]

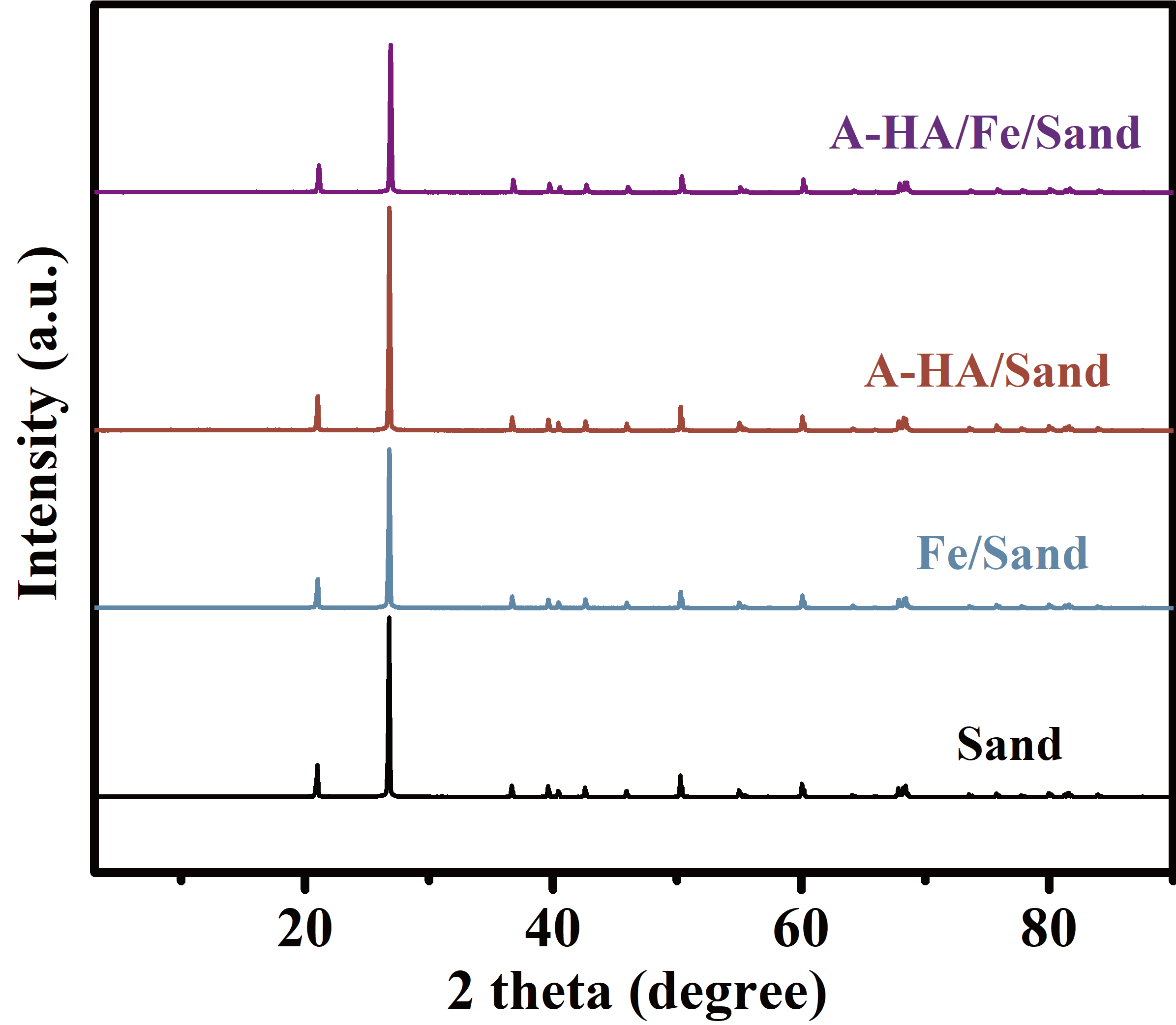

Supplement: Supplementary 1 — Supplementary Text S1 to S3 Tables S1 to S5 Figs. S1 to S5 [file research.0308.f1.zip › Figure S5.png]
